# Adapting and deploying a digital program for training non-specialist providers on a brief psychological intervention for depression in rural Gujarat, India

**DOI:** 10.1371/journal.pgph.0003967

**Published:** 2024-12-05

**Authors:** Aakrushi Brahmbhatt, Darshana Rathod, Udita Joshi, Azaz Khan, G. Sai Teja, Shrey Desai, Ajay Chauhan, Shobha Shah, Deepti Bhatt, Sethuraman Venkatraman, Deepak Tugnawat, Satchit Balsari, Vikram Patel, Anant Bhan, John A. Naslund

**Affiliations:** 1 Sangath Bhopal Hub, Bhopal, Madhya Pradesh, India; 2 Society for Education Welfare and Action-Rural, SEWA RURAL, Jhagadia, Bharuch, Gujarat, India; 3 State Mental Health Authority, Ahmedabad, Gujarat, India; 4 Hospital for Mental Health, Ahmedabad, Gujarat, India; 5 Argusoft India Ltd, Gandhinagar, Gujarat, India; 6 Department of Global health and Population, Harvard T.H. Chan School of Public Health, Boston, MA, United States of America; 7 Department of Global Health and Social Medicine, Harvard Medical School, Boston, Massachusetts, United States of America; Dalhousie University, CANADA

## Abstract

Workforce shortages represent a major bottleneck to delivering depression care, particularly in lower resource settings. This pilot study aimed to assess the acceptability and feasibility of a digital training program on developing knowledge and skills in the delivery of a brief behavioral activation intervention for depression among non-specialist providers (NSPs) in Gujarat, India. Participating NSPs, such as community health workers and other frontline providers without specialized training in mental health care, were provided access to a digital program covering the core skills and content necessary to deliver the Healthy Activity Program, an evidence-based behavioral activation intervention for depression. NSPs completed knowledge assessments before and after the digital training, followed by focus group discussions to gather their feedback about the program content and delivery format. Of 43 NSPs enrolled in this study, 67% (n = 29) were community health workers called Accredited Social Health Activists and 33% (n = 14) were frontline mid-level health providers called Community Health Officers. Most participants (n = 39; 91%) completed the full course. Knowledge assessment scores showed improvement from pre-training (mean = 29.96; 95% CI: 27.12–32.81) to post-training (mean = 34.62; 95% CI: 31.05–38.19; p = 0.0448). Focus group discussions revealed that participants appreciated the digital mode of training despite facing technical challenges while completing the course. This study further supports the feasibility and acceptability of digital approaches for training frontline providers to deliver brief psychological interventions for depression. With adequate resources and proper execution, digital training holds potential to serve as a key tool to build capacity of NSPs and expand the mental health workforce in India.

## Introduction

Depression is a leading cause of morbidity affecting roughly 5% of the adult population globally [1]. This equates to approximately 280 million people living with depression, most of whom reside in lower income countries [1]. Over 75% of people living with depression in low-income and middle-income countries (LMICs) do not have access to any mental health care [1]. The World Health Organization (WHO) estimates that mental health conditions will contribute to economic losses exceeding 1.03 trillion USD from 2012–2030 [2]. Specifically, in India, it is estimated that 1 in 7 adults experience mild to severe psychiatric ailments [3], with lifetime prevalence and current prevalence of depressive disorders of 5.3% and 2.7%, respectively [4]. In Gujarat, the target setting for this study, the prevalence of depressive disorders has been reported between 1.2% [5] and 3.3% [3].

Although the Indian government has adopted new policies and legislations such as the National Mental Health Program (1982), the District Mental Health Program (1996), and the Mental Health Care Act (2017), ensuring accessibility and availability of mental health services remains a significant challenge [6, 7]. A large treatment gap persists due to inequities and inefficiencies in resource allocation [8]. There is also a chronic shortage of specialist mental health professionals and health care facilities equipped with skills in responding to the mental health needs of their patients. The few health facilities with available mental health services that do exist are typically concentrated in urban areas, leaving many individuals in rural areas without adequate access to mental health care [9]. Low health literacy, lack of health seeking behaviors, and negative attitudes around mental disorders in the form of stigma also play a role in preventing people from getting help [10, 11].

To mitigate these barriers, the delivery approach known as ‘task sharing’ has gained attention, where non-specialist community health workers are equipped with counselling skills necessary for responding to a range of mental disorders [12, 13]. The WHO recognizes task-sharing as the rational redistribution of tasks among health workforce teams [14]. This approach involves training and building capacity of frontline providers, who have not previously completed any specialized mental health training, to take on roles of delivering mental health care [12]. The delivery of effective psychosocial interventions for mental disorders can be assigned to non-specialist providers (NSPs) as a means to increase mental health care coverage in both low and high-income settings [15]. To successfully implement task-sharing models of mental health care delivery, it is necessary to first train and build capacity of NSPs. To date, conventional methods for training frontline NSPs pose challenges to achieving scale given the reliance on classroom-based instruction, which represents the most widely employed method of health worker training in LMICs. This approach is also costly given the continued need for mental health specialists to facilitate these trainings, travel costs of NSPs to reach the designated government health centers for attending the trainings, and additional logistical resources for coordinating the trainings. Therefore, training NSPs at scale represents a key bottleneck to implementing task-sharing models.

Digital tools (e.g., websites, mobile applications) may yield opportunities to overcome the logistical challenges of scaling up training and support for frontline health workers to facilitate task-sharing in low-resource settings. Broadly, there has been considerable interest in the ways digital technologies could be used to advance mental health care services in low-resource settings [16–19]. In India there have been numerous promising studies leveraging widely available digital technology to support and train frontline health workers [20–23]. Of relevance to the current study, the TeCHO platform has been used in Gujarat state since 2018 to improve comprehensive primary care services through real-time data entry and the generation of alerts for high-risk cases, supportive supervision, and report automation at multiple levels [24, 25]. Recent studies have also demonstrated that use of the TeCHO platform among frontline health workers has been successful in improving access to maternal and child healthcare services, as well as improved quality reporting and monitoring practices in Gujarat [26]. Furthermore, the cost-effectiveness of the TeCHO platform has been demonstrated, where the incremental costs are offset through averted disability attributed to the early identification of high-risk cases among pregnant women and children [27]. Such examples show the potential for mobile health (mHealth) tools to streamline and facilitate health care delivery processes.

More recently there have been efforts to use digital technology for training community health workers (CHWs) in the delivery of mental health interventions [28, 29]. This includes the development of a digital program for training CHWs in Sehore district in Madhya Pradesh in the delivery of a brief psychological intervention for depression called the Healthy Activity Program (HAP) [30–34]. The HAP digital training focuses on the knowledge and skills required to deliver HAP, a counselling intervention for mild to moderate depression based on behavioral activation and adapted for use in India [35]. The acceptability and effectiveness of the digital training were reflected in a pilot study followed by a randomized controlled trial enrolling frontline health workers in Madhya Pradesh [32, 34]; yet, the training content has not been deployed in other regions or states in India, representing a critical next step towards achieving scale. Additionally, in other parts of India, technology has been successfully deployed for training primary care doctors in identifying and responding to mental health needs in rural settings [36, 37].

This study seeks to expand upon this recent work with the overall aim of adapting the HAP digital training program for depression care for use in primary health care settings in Gujarat. This study involved leveraging the established reach of the TeCHO platform in Gujarat to serve as the digital platform where frontline health workers could access the HAP digital training program. The HAP course was hosted on the Learning Management System (LMS) within the TeCHO platform which delivered the course through various videos and engaging quizzes. This involved taking the digital training content previously developed and deployed in Madhya Pradesh, translating the content for use in Gujarat, and then uploading it onto the TeCHO platform accessible through a smartphone app. The objective of this study was to assess the impact of the digital training program on participating health workers’ knowledge scores, as well as their satisfaction with the adapted digital training program in a new setting.

## Methods

### Ethics statement

Ethical approval was obtained from the Institutional Review Boards of Sangath and the Society for Education, Welfare and Action-Rural (SEWA-Rural). Sangath is a not-for-profit research organization that has been working in the field of mental health since 1996. SEWA-Rural, established in 1980, is a community-based organization that works to improve the health and welfare of people in the tribal block of Jhagadia in the southern part of Gujarat. All non-specialist providers (NSPs) in this study provided written informed consent according to procedures approved by the Institutional Review Boards of Sangath and SEWA-Rural.

### Setting

This mixed-methods pilot study was conducted in the Jhagadia block of the Bharuch district in Gujarat, India. Gujarat is a state located on the western coast of India and has a population of over 60 million people, according to the 2011 census [38]. Bharuch is a predominantly rural district in southern Gujarat with over 1.5 million inhabitants of which roughly 66% reside in rural areas [39]. There are four psychiatric hospitals across the entire state of Gujarat, all of which specialize in long-term treatment of mental illnesses. Gujarat is also home to innovative community mental health programs including Dava-Dua, QualityRights, and Atmiyata [40–42]. Despite these community-based efforts, there remain many challenges around creating accessible mental health services at the primary health care level, driven largely by inadequate workforce capacity, and lack of specific training in delivery of proven mental health interventions.

### Participants

This study conducted in Bharuch, Gujarat, employed a purposive sampling technique and the target sample consisted of non-specialist providers (NSPs) without any prior formal training in mental health care delivery, including CHWs referred to as Accredited Social Health Activists (ASHAs) and frontline mid-level health providers referred to as Community Health Officers (CHOs). The CHOs are more recently deployed mid-level health providers, introduced as part of India’s Comprehensive Primary Health Care reforms under the Ayushman Bharat program to enable delivery of an expanded range of comprehensive primary health care services within Health and Wellness Centers [43, 44]. Health and Wellness Centers are upgraded primary health facilities that offer preventive, promotive and curative health care services. CHOs, often primarily AYUSH or nurse practitioners who have undergone additional training, are deployed as the leaders of Health and Wellness Centers. The eligibility criteria for NSPs in this study included being 18 years or older, employed by the National Health Mission, and recommended by the state Ministry of Health and Family Welfare for participation in the study.

### Study procedures

The list of eligible NSPs was provided by the Taluka Health Officer and approved by the Chief District Health Officer as per the directives of the National Health Mission in Gujarat. All nominated NSPs were invited for the recruitment information session and represented three distinct groups based on their cadre (i.e., type of health worker), and prior exposure to the TeCHO platform. The first group consisted of ASHAs who had Android mobile devices provided by the state health department and were using the TeCHO platform. The second group consisted of ASHAs who did not have any prior exposure to the TeCHO platform and were provided Android mobile devices from Sangath to use for the study duration. The final group consisted of the CHOs who owned their own Android mobile devices and were using the TeCHO platform. As part of the information session, interested NSPs could then choose to enroll in the study. All NSPs completed written informed consent according to procedures approved by the Institutional Review Boards of Sangath and SEWA-Rural prior to enrolling in the study. After completing informed consent, all participants attended an orientation for the training program, introduction to use of the TeCHO digital platform, and collection of baseline measures of knowledge. The group of ASHAs that did not have prior experience with the TeCHO platform received additional training on the use of digital devices to overcome potential digital literacy challenges and to facilitate their use of the digital training content. Participant recruitment and data collection was completed from May 1^st^, 2021 to August 31^st^, 2021.

### Digital training program and adaptation for use in Gujarat

A digital training program covering the HAP content was originally developed for use in Madhya Pradesh by adapting and transforming in-person instruction approaches, such as role-plays, into digital content that could be accessed from a smartphone app. This process of developing the digital training has been previously described in detail [30, 45]. HAP is a brief psychological intervention for depression that is delivered over 6–8 sessions and centers around behavioral activation as the core psychological framework with added emphasis on problem-solving strategies and the activation of social networks [35, 46]. The digital course includes 18 modules with roughly 100 videos, presentations, reading materials, engagement quizzes, and assessment questions, requiring about 50–60 hours to complete [30].

In the current study, this existing digital training program was adapted for use in Gujarat. This involved translating the content into the local language, Gujarati, and adapting the digital content so that it could be hosted onto the TeCHO smartphone app via a boutique newly developed Learning Management System (LMS) integrated into the application. Argusoft India Ltd, who had developed the TeCHO App also developed the LMS for TeCHO. TeCHO is a mobile and web-based application that enables data entry by the person providing services at the time and place of service delivery [24]. The digital course for our study was developed through the contextualization and adaptation of the existing HAP course in Hindi, previously developed and tested among NSPs in Madhya Pradesh [30]. Contextualization of the course content for use in Gujarat involved translation, adaptation, and fidelity checks. The entire HAP manual along with all other resources and video scripts were first translated into Gujarati, the local language. A research team member with knowledge of both the local language and content cross-checked and approved the final version of the course content. Stakeholder workshops were convened, consisting of small groups of 3–5 health workers, initially after translation of all materials and then again after the instructional videos were filmed. Stakeholder feedback primarily concerned the clarity of terms used, the need for correct use of local language, and modifying the context in role-play scenarios. These recommendations were incorporated into the program and the final content was reviewed by the research team prior to being uploaded to the digital training LMS platform, which was then shared with the NSPs.

**Fig 1:**
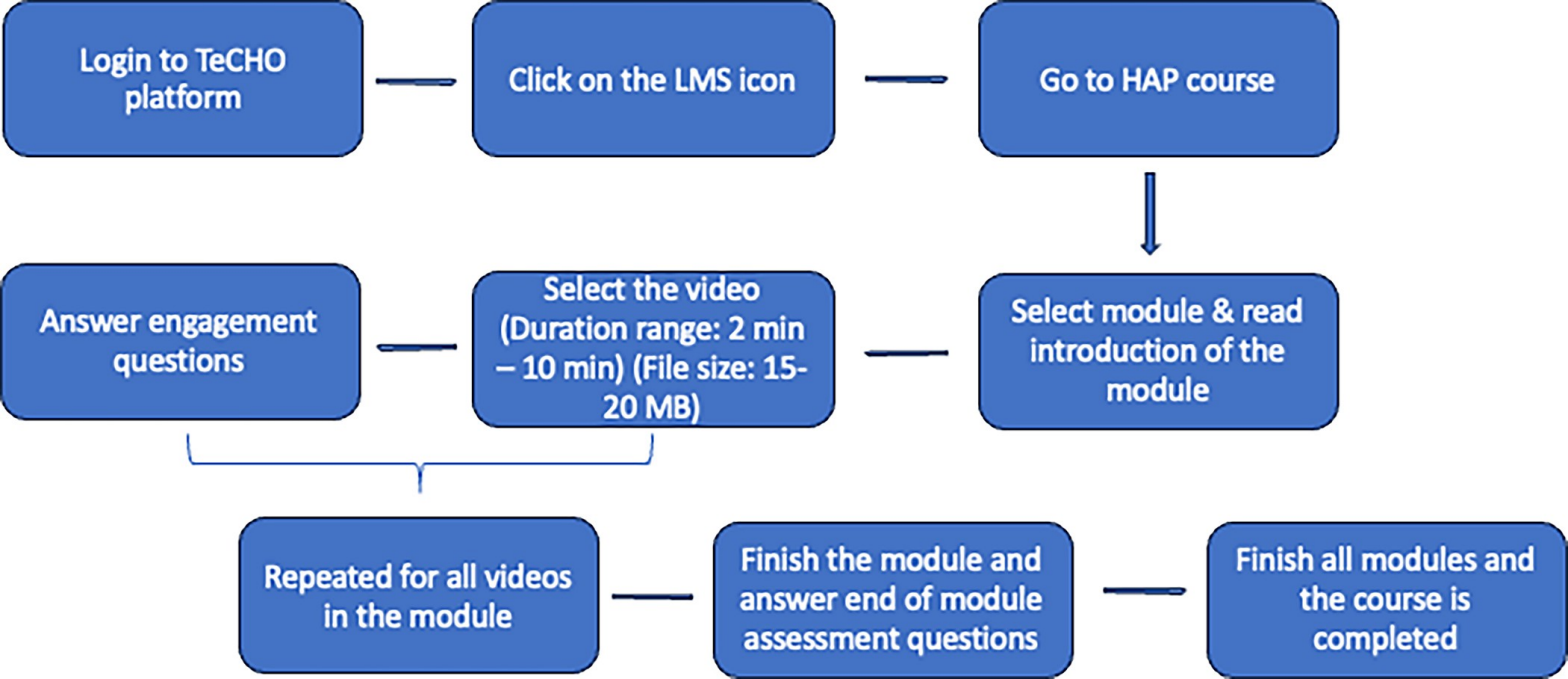
TeCHO Learning Management System pathway for learners.

Participants had 21 days (3 weeks) to complete the digital training. The research team provided technical support to participants as needed throughout the training, primarily through regular check-ins and by being available for troubleshooting by phone or over WhatsApp. The process for accessing the training on the TeCHO app is outlined in Fig 1. The research team tracked the progress of all the participants with the help of a dashboard accessible only to the research team. This highlighted the participants with slow or no progress. Those participants were contacted by the research team via calls and were offered encouragement to complete the course along with support with troubleshooting various technical challenges faced, if any. A WhatsApp group among participants and the research team also helped to facilitate regular communication, and to share updates, and supporting mitigation of any potential challenges.

### Outcome assessment

The main outcomes were the acceptability and feasibility of the adapted digital training program, assessed based on completion rates of the training, completion of a post-training satisfaction questionnaire, and feedback provided through in-depth qualitative focus group discussions. Additionally, we assessed the preliminary effectiveness of the adapted training program by measuring change in scores on a written competency assessment among participants. Data collection occurred at baseline (pre-training) and endline (post-training). Demographic characteristics were also collected at baseline for all participants.

### Knowledge assessment questionnaire

The primary effectiveness outcome was assessed with a validated knowledge questionnaire consisting of 26 multiple-choice questions [47]. The questionnaire evaluates change in participants’ applied knowledge and skills about the HAP intervention after completion of the digital training program. The questionnaire uses short-case vignettes followed by multiple-choice questions, and was developed and adapted for use in Madhya Pradesh [47, 48]. This measure was translated into Gujarati, and each correct answer is considered one point, meaning higher scores indicate greater knowledge of the HAP intervention for depression. The scores were then converted to 0–100 scale to facilitate interpretation. This measure was collected pre- (baseline) and post-training (endline).

### Attitudes about mental illness

The Knowledge, Attitudes and Behaviors (KAB) questionnaire captures participants views about mental illness, and has been used previously with health workers in India [49, 50]. The KAB measure was translated into Gujarati and was collected pre- (baseline) and post-training (endline).

### Satisfaction questionnaire

The satisfaction questionnaire was adapted from the MUSIC model [51], a measure composed of 26 questions, and was translated into Gujarati. The measure captures data on various key constructs pertaining to acceptability of the digital training, including empowerment (i.e., the individual has control over his/her learning environment in the course), usefulness (i.e., the course content is useful for his/her future), success (i.e., he/she can succeed at completing the digital course), interest (i.e., the instruction and course content is interesting), and caring (i.e., the instructor cares about whether students succeed in completing the course) (Jones 2019). Participants rate each item on a Likert scale, ranging from 1–6, where 1 is the lowest and 6 is the highest score. The items are scored across each of the key constructs, and this measure was collected post-training.

### Qualitative focus group discussions

Participants were invited to join focus group discussions after completing the training. Three focus group discussions were conducted based on participants familiarity with TeCHO as a means to collect targeted feedback, and ensure all participants would have the chance to share their views about the program. The first group consisted of ASHAs who were using TeCHO in their routine work, a second group consisted of ASHAs without any experience of using TeCHO, and the third group included CHOs. Participants shared their insights about their experiences with the training, with emphasis on the acceptability of the digital platform and course content and offering recommendations for improvement. The focus group discussions lasted about 60 minutes and were audio-recorded and transcribed for thematic analysis.

### Data analysis

Participant demographic characteristics were summarized using descriptive statistics. The primary effectiveness outcome was the multiple-choice measure of applied knowledge, where pre-post changes scores were analyzed using paired *t* tests. Wilcoxon sign paired rank test was used to test the null hypothesis that the post-training knowledge scores will be higher than pre-training knowledge scores. Pre-post differences in scores on the secondary KAB measure was analyzed using the non-parametric test (Wilcoxon sign paired rank test). The satisfaction questionnaire responses were tabulated and summarized in a table. All statistical analyses were completed using Stata version 17.0 and *p*<0.05 was considered statistically significant. Qualitative data on program acceptability and feasibility obtained from the focus group discussions were analyzed thematically. Focus group discussions were coded based on pre-defined themes pertaining to benefits, challenges, and recommendations for improving the training program, allowing for flexibility for additional themes to emerge from the codes [52]. An external researcher who was not part of the development or delivery of the digital training led the thematic analysis to minimize risk of bias [52]. One individual read and coded the transcripts, and then the research team met to review the codes and key themes, which offered a chance to resolve any disagreements and reach consensus.

## Results

In total, 45 non-specialist providers (NSPs) were nominated by the district, of which 43 consented and enrolled in the study to complete the digital training. Table 1 lists the demographic characteristics of participating NSPs, consisting of 28 ASHAs, 1 ASHA Facilitator (a senior ASHA worker, who supervises around 20 ASHAs each along with providing community care), and 14 CHOs. Participants’ mean age was 33.3 (SD = 7.9) years, and most participants were female (93%). Nearly all (98%) participants reported having used a smartphone previously, with most (86%) indicating that they owned a smartphone. A total of 39 participants (91%) completed the entire course, requiring between 12 and 21 days of time. We did not observe any statistically significant differences in training completion rates or outcomes between participants with prior experience using the TeCHO platform compared to those without prior experience using the TeCHO platform.

**Table 1:** Sociodemographic characteristics of participating non-specialist health workers.

Participant Sociodemographic Characteristics		
Group	Numbers (n = 43)	Percentage (%)
ASHA	28	65.1
ASHA Facilitator	1	2.3
CHO	14	32.6
**Age in years (Mean, SD)**		
Mean	33.3 (7.9)	
**Gender**		
Male	3.0	7
Female	40.0	93
**Education**		
8th - 9th	5	11.6
10th	13	30.2
12th	10	23.3
Graduation & above	15	34.9
**Work experience in months (Mean, SD)**		
Mean	71.1 (56.0)	
**Ownership of mobile device**		
Yes	37	86
No	6	14
**Type of mobile device** (Among those who own mobile) (n = 36)		
Smartphone	32	88.9
Other	4	11.1
**Experience using smartphone**		
Yes	38	88.4
No	5	11.6
**Size of the household**		
Mean	5 (1.7)	

### Preliminary effectiveness of the digital training

Improvements in applied knowledge scores were observed from pre-training (mean = 29.96; 95% CI: 27.12–32.81) to post-training (mean = 34.62; 95% CI: 31.05–38.19). Based on the Wilcoxon sign paired rank test, the pre-post change observed in the competency test score was statistically significant (p = 0.045).

### Attitudes about mental illness

Participants’ median score was 42 pre-training on the KAB measure, and increased to 43 at the post-training assessment, though this change was not statistically significant based on the Wilcoxon sign paired rank test (p = 0.74). These results are outlined in Table 2.

**Table 2:** Change in participants’ knowledge, attitudes and behaviors questionnaire responses from pre-post training.

Domain	Baseline				Endline			diff	Wilcoxon sign rank test (p-value)
	n	Mean	Median	SD	Mean	Median	SD		
Knowledge	39	13.26	13.00	2.10	13.03	13.00	2.24	-0.2	0.8055
Attitudes	39	14.02	14.00	2.65	13.95	14.00	2.25	-0.1	1
Behaviors	37	15.80	16.00	2.58	16.03	16.00	2.21	0.2	0.2322
Overall	36	43.25	42.00	5.72	43.14	43.00	5.14	0.11	0.7406

### Satisfaction questionnaire

The satisfaction questionnaire responses were generally quite high, with most key constructs on the measure scoring at least 5 or greater out of a 6-point Likert scale. Specifically, the empowerment (mean = 5.02; 95% CI 4.76–5.29), usefulness (mean = 5.29; 95% CI 5.04–5.53), success (mean = 5.03; 95% CI 4.78–5.27), interest (mean = 5.02; 95% CI 4.83–5.21), and caring (mean = 5.00; 95% CI 4.75–5.24) components of the measure each scored 5 or greater.

### Acceptability and feasibility of the digital training

Thematic analysis from the focus group discussions revealed 5 broad themes, including: satisfaction with the training program; benefits of the training program; challenges faced and what participants did not like about the training program; navigation issues with using the training app; and recommendations for improving the training and the digital training app. These broad themes, sample codes, and representative quotes are summarized in Table 3.

**Table 3:** Overarching themes about the feasibility and acceptability of the digital training program from the focus group discussions.

*Theme*	*Representative Quotes*
**Overall satisfaction with the digital training program**	*‘Through the videos and books*, *we got to know more about mental health*.*’(ASHA worker familiar with TeCHO platform)*
	*‘When we came here for the first time*, *we thought that we might also come under depression*. *We thought that how will we cover so much course*? *We enjoyed watching more than reading’*. *(ASHA worker familiar with TeCHO platform)*
	*“We understood that the training was about mental treatment*. *We have to understand the patient’s talk*, *how to provide counselling*, *what help can he get from us*, *counselling and treatment for mental diseases*.*” (ASHA worker new to TeCHO platform)*
	*“How to take the session*, *in how many parts*, *what are the stages of treatment*, *how to give the treatment like this in every video*, *every chapter was there*.*” (ASHA worker new to TeCHO platform)*
	*“It is boring to learn everything at once but with videos*, *one can watch for some time and take a break and watch whenever they want to*.*” (ASHA worker familiar with TeCHO platform)*
	*“If someone is ill*, *they can’t come for the training*. *But with digital they can watch it at home*.*” (ASHA worker new to TeCHO platform)*
**Benefits of the training and how it can help them (self and work)**	*“I am an ASHA worker and training helped me in a big way*. *The training helped me to deal with my alcoholic husband*. *I showed my husband the videos on alcohol addiction*. *Although he was resistant to watch the videos with me*, *I coaxed him to watch them in order to help me in answering the questions at the end*. *After this*, *his addiction hasn’t stopped but at least he has stopped harassing me and my kids like he did before*. *My mental stress has improved a bit now*. *He has improved 25% and I am confident that the remaining 75% will also improve soon*.*” (ASHA worker)*
	*“Till now we had the training face to face*, *this was the first time that we had to go home and watch the videos and then answer the questions……*. *this was the only training where we could watch the videos at our time and do our work at our time*.*” (ASHA worker new to TeCHO platform)*
	*“The role-plays were good*. *They could be easily remembered*.*”*
	*(ASHA worker new to TeCHO platform)*
	*“We can encourage family members to take the person with mental health issues to a hospital rather than a tantric*.*”*
	*(ASHA worker familiar with TeCHO platform)*
	*“In this mental health training*, *we learned that we can also help people in our family who might be suffering from these problems and at least help them come out of it*.*” (CHO)*
	*“We will gain more trust of the community in the field*. *If we identify 1 or 2 patients in the field and give treatment to them*, *then people can also understand that this kind of treatment is available*.*” (ASHA worker familiar with TeCHO platform)*
**Challenges faced and what they did not like with respect to the digital training experience**	“*There were network issues*. *Due to that it was getting refreshed constantly and the quiz was gone*.*” (CHO)*
	*“We have to refresh more*. *After finishing the video*, *you can’t attend the quiz*. *We have to press back*, *refresh it and then only quiz can be attended*. *So*, *we have to refresh it once after watching one video*, *and then only it moves forward*.*” (CHO)*
	*“Secondly*, *there were some files in which there were error or crash files*. *So*, *when it was connected to phone*, *you had to delete it*, *find it and then download it again and it used to crash sometimes so that was problematic*.*” (CHO)*
	*“It also happens that while watching the video*, *it stops and then if we start it*, *it starts from the beginning*. *If we try to forward that*, *then also it shows that you have to watch the video from the beginning*.*” (CHO)*
	*“Some of the videos were very long*.*” (ASHA worker familiar with TeCHO platform)*
**Navigation issues of the training app used**	*“The platform is good all the way*. *When we first came*, *we thought what is this and what will they do and we felt that this might be something different*. *But then they explained about the project in detail and about the training and about the 21 days training at home*, *so we understood that this was good*.*” (CHO)*
	*“When one video was finished*, *then only second video would begin*. *A word*, *“WATCH” would come*, *followed by question answers with the options and then only second video would open*. *One could not skip the videos*. *To answer the questions*, *it is necessary to watch the video*.*” (ASHA worker familiar with TeCHO platform)*
	*“We could give the answers after watching the videos*, *so it was good*.*” (ASHA worker new to TeCHO platform)*
	*“In that*, *there were three attempts*. *So*, *if first is wrong*, *then second and third attempt was coming right*, *so no issues*.*” (CHO)*
	*“I like the videos*. *That was a good method*. *The explanation was easy and the speech was also clear*.*” (CHO)*
**Suggestion to improve the digital training**	“*Make the videos available offline*, *then you can download and watch it anytime*.*” (CHO)*
	*“If there is an option where if the video is of 15 minutes*, *and we can pause it at 3 minutes and then if we come back and start the video from there itself*, *it will be good*. *Currently also there is the option of pause but we have to restart the video*. *Then we can start from where we stopped*.*” (CHO)*
	*“Some videos were long*. *Length of a video should be around 4–5 minutes*.*”*
	*(ASHA worker familiar with TeCHO platform)*
	*“There should be a supervisor*. *If we try to explain something to someone and they don’t understand*, *the supervisor can come and explain*. *So*, *if 2 to 3 people tell the patient*, *he might realize that this is important*.*”*
	*(ASHA worker new to TeCHO platform)*

Participants shared positive views about the digital training and commented on aspects they appreciated including convenience of time, feasibility, and the video-based content. Participants enjoyed the visual and audio quality of the videos and found that they could easily understand the issues discussed as it was in Gujarati. Participants preferred the video format over reading material; yet, the reading material was viewed as important understanding and reviewing difficult concepts. They also highlighted that HAP would be useful in their communities and for themselves (mental health self-care). Participants expressed appreciation for the digital training in comparison to face-to-face conventional training programs. They also highlighted a few key benefits of the digital training including, feasibility in terms of flexibility to take course at a time of their choice, saving of resources for travel to central training facilities, and self-paced learning that facilitates more retention and makes it possible to revisit the content at their own leisure. Participants also highlighted challenges they faced during the training, such as technical errors due to application insufficiencies and internet bandwidth issues. In terms of navigating the content in the app, participants explained that they got used to it after a point of time. While mostly enjoying the videos, some participants remarked that some videos seemed lengthy or repetitive, and they offered suggestions about videos that should be shortened.

Participants suggested having an in-person orientation before beginning the digital training to address technical issues and become familiar with the app. Participants also emphasized the importance of completing the training and mentioned the value of having family support to enable them to do the training. These participants mentioned that help with household chores and childcare that family members offered could free up more time to enable them to finish the training as typically they took the course during morning or evening hours. Several ASHAs highlighted the role of having a supervisor to assist with responding to the needs of patients. The CHOs’ suggestions were more inclined towards the technical aspects, as they shared recommendations to make the videos available offline to address low bandwidth, and taking care of connectivity and platform issues such as refreshing and downloading the content.

## Discussion

This study assessed the acceptability and preliminary effectiveness of a digital program for training frontline health workers to deliver a brief psychological intervention for depression adapted for use in Gujarat, India. Participants showed improvement in their scores on a validated measure of applied knowledge, suggesting the training was successful in teaching NSPs the skills and applied knowledge necessary for delivering the intervention. Participants showed no change in their attitudes about mental illness, suggesting that perhaps these health workers were sensitive towards mental health before enrolling in the study, and therefore, held fewer misconceptions about mental health concerns. In terms of qualitative feedback, through focus group discussions, participants highlighted their satisfaction with the training, and how the training was helpful, and expressed their preference for digital compared to conventional in-person training. They also commented on various challenges they faced when using the digital platform and provided constructive suggestions for improving the program.

Our study adds to emerging evidence showing that digital technology can support health worker training in LMICs [53]. In terms of gaining the necessary applied knowledge and skills to deliver brief psychological interventions, digital training appears to be as effective as traditional classroom or face-to-face training [32, 54, 55]. Consistent with prior studies, we found that participants favored the digital mode of training as it is more convenient, and found the digital training [31] content to be a new and interesting to learn. Furthermore, participants appreciated the flexibility and convenience of the digital training in terms of time, place, and avoiding the need for transportation to designated classroom-based facilities. In a recent study, training videos were also considered useful as they offer an audio-visual demonstration of psychological concepts [28]. Use of captivating clip-art images in videos and quizzes, with the addition of interactive role-plays, proved useful techniques as they were quickly accepted and helped participants to learn the new material and concepts. Participants also mentioned that the addition of the printed materials was also valuable for learning the different concepts. These findings are consistent with prior research showing that interactive, rich media can encourage participant engagement with training materials [56].

ASHAs’ limited prior experience using smartphones, length of the videos, poor internet connectivity, limited device storage space, certain navigation features of the TeCHO app, and technical glitches posed challenges in accessing and completing the training. Some participants were not pleased with certain mandatory features of the app requiring them to watch videos. Similar technological drawbacks were observed in a previous study [57]. For participants who were not well versed with smartphones, it was a challenging task to navigate the app, highlighting the need for orientation and targeted assistance. This kind of issue was noted in a prior study with frontline health workers in Ethiopia [57]. Importantly, our team has since modified the training content to better function in settings with lower bandwidth, by reducing the length of the video-based content, and ensuring smaller file sizes to avoid delays with loading [30]. Furthermore, since launching this study, there have been significant advances in terms of the access and quality of digital devices, particularly in rural settings in India. This is reflected by the improved connectivity and bandwidth in rural areas, increasing access to smartphones among frontline health workers that was further accelerated in the aftermath of the pandemic, and greater familiarity with using digital devices for engaging in training and supporting other work-related activities [58–60]. Therefore, while we noted a range of technical challenges in this study, these do not represent insurmountable barriers to employing the use of digital training for building workforce capacity among rural frontline health workers at scale.

Participants offered suggestions, including the need to reduce the length of several videos, pre-download the video files to the devices to address buffering issues, make technical support available, and improve the functioning of the app to make the digital training more user-friendly. Consistent with prior research, there is a need to engage end users early in the design to promote adoption and use of digital interventions to manage NCDs such as depression [61]. Prior similar efforts have studied task-shifting and mobile technology-based electronic decision support systems as approaches for enhancing the skills and abilities of primary care health workers. One study trained frontline providers to provide evidence-based mental health care for stress, depression, and suicidal risk in 30 remote villages in the Indian state of Andhra Pradesh [62]. Participants in this prior study found the digital training satisfactory and informative as it helped them to understand the fundamentals of mental health in an interactive way. Consistent with the current study, the digital training appeared to equip frontline providers with the skills to screen and identify individuals from their communities struggling with mental illness. Another study showed that frontline health workers can play an essential role in providing psychological interventions in the community [63].

The COVID-19 pandemic was a major driving factor for the adoption of digital technology [58, 60], even in rural India. The Health and Wellness Centers are now expected to use technology to address healthcare delivery and staff shortages. Due to drug shortages, travel restrictions, and financial hardships, many patients experiencing mental health concerns were unable to receive necessary care. Teleconsultations enabled healthcare providers to ensure continuity of care. The Indian government’s launch of the eSanjeevani OPD during the COVID-19 pandemic was critical and timely; yet, patients’ main challenges appeared to be related to their low levels of education and computer literacy, and language barriers [64], highlighting the need for connection with community-based providers for delivery of mental health services. As the National Health Authority considers expanding digitization of healthcare services across India through the Ayushman Bharat Digital Mission [65], it is important for the state to invest in internet availability at the sub-center and primary care center level, where the need for tele-services is even more salient. It may also be advisable, until universal broadband penetration is received, to design tools that allow for asynchronous, offline, operations. Furthermore, programs at the health system-level aimed at equipping frontline health workers with digital tools to support them in their work represent key opportunities to support the uptake of digital training programs and to ensure the sustainability of such efforts [19, 66, 67]. Our findings indicate that digital training in rural and underserved areas, which if scaled up, can be a potentially sustainable and time-saving solution for building capacity in the delivery of evidence-based mental health services. Efforts to leverage digital training program not only hold promise for training and supporting NSPs, but could support cost-effective and scalable approaches for training other health care providers, including primary care doctors and nurses [36, 37].

This pilot study is limited in terms of generalizability as it was conducted in one block of a rural district of Gujarat and a purposive sampling technique was used to identify participants. The sample size was small, with a total of 43 participants, which makes it difficult to draw conclusions about the broad impact of the training. With the small sample size, it was also not possible to determine if any participant characteristics might have influenced study outcomes, such as digital literacy or prior exposure to the TeCHO digital platform. Technical problems encountered during the digital training, including poor internet connectivity, application server issues, and storage limitations on the digital devices represented key issues to address to scale up such training efforts. The COVID-19 pandemic posed challenges during the training implementation, requiring adoption of enhanced safety protocols for data collection [68]. Lastly, this study is limited because it did not involve collecting data on the newly trained NSPs’ quality of HAP delivery in routine care settings, thereby highlighting a critical next step for advancing this work. Furthermore, this study did not include longer-term follow up to better understand whether the knowledge gained from completing the training had other impacts on the day-to-day work activities and performance of the participating NSPs. Future studies are needed to consider how digital training programs like the one evaluated in this study may result in changes in practice or application of new knowledge in the field among frontline NSPs.

## Conclusion

This study adds to mounting evidence on the acceptability and effectiveness of digital programs for training NSPs in the delivery of proven mental health interventions, such as HAP. These findings highlight that the digital training content can be adapted for different settings and hosted on diverse digital learning management system platforms, in this case the TeCHO platform, demonstrating the potential to scale this training program across different regions of India and globally. This digital training program holds potential to help build capacity of NSPs in India and make mental health care more accessible in underserved areas [69].

## Supporting information

S1 Checklist Inclusivity in global research questionnaire. (PDF)

S1 Data Baseline results. (CSV)

S2 Data Endline results. (CSV)
